# Supplementary material for: A simple method to estimate flow restriction for dual ventilation of dissimilar patients: The BathRC model
Source: PLoS One. 2020 Nov 16;15(11):e0242123. doi: 10.1371/journal.pone.0242123 (PMC7668571; doi:10.1371/journal.pone.0242123)
Supplement: S1 File — Appendix A. (DOCX) [file pone.0242123.s001.docx]

**Appendix A**

As shown in Figure 1, the model consists of the patient lung/airway resistance (*R*) and compliance (*C*), and a resistance (*R_v_*) and compliance (*C_v_*) representing the properties of the ventilator tubing system. The airway flow rate*, q*, is related to the rate of change in airway pressure *p_p_*, according to:

| $R\frac{dq}{dt}+\frac{1}{C}q=\frac{dp_{p}}{dt}$ | (1) |
| --- | --- |

And flow from the ventilator, given by tubing pressure drop divided by resistance, is split between the patient and what is absorbed by tube compliance (including gas compressibility):

| $\frac{p-p_{p}}{R_{v}}= q+C_{v}\frac{dp_{p}}{dt}$ | (2) |
| --- | --- |

Combining equations (1) and (2), the gas volume reaching the patient’s lungs, *v*, can be related to the ventilator pressure, *p*, by:

| ${RCR}_{v}C_{v}\frac{d^{2}v}{dt^{2}}+\left( {RC+R}_{v}C_{v}+R_{v}C \right)\frac{dv}{dt}+v=Cp$ | (3) |
| --- | --- |
|  |  |

Physically realistic parameter values lead to a very small second derivative term, so:

| $\left( {RC+R}_{v}C_{v}+R_{v}C \right)\frac{dv}{dt}+v\approx Cp$ | (4) |
| --- | --- |

It can be shown that the system in Eqn. 3 is always overdamped for physically possible values of $R$, $C$, $R_{v}$ and $C_{v}$ (i.e. positive values). The second order system is thus comprised of two first-order lags. Using the nominal parameter values from the experiments below, these lags have time constants of 1.25 s and 0.0296 s. For comparison, the first order system in Eqn. 4 has a single time constant of 1.28 s. This value is very close to one of the second order time constants, while the remaining time constant is too small to have a significant contribution at the timescales considered. Transfer functions for the first-order system and the second-order system differ by less than 2% in amplitude at the nominal respiration rate of 0.25 Hz.

Due to ventilator or patient properties, the inspiration and expiration parameters may be different. Therefore, the first order response represented by (2) may have different inspiration and expiration time constants, given by:

| $\tau_{i}={R_{i}C_{i}+R}_{vi}\left( C_{vi}+C_{i} \right)$  $\tau_{e}={R_{e}C_{e}+R}_{ve}\left( C_{ve}+C_{e} \right)$ | (5) |
| --- | --- |

In pressure control, the ventilator pressure *p* approximates a series of square pulses. The lung volume during inspiration at time *t*, in addition to the Functional Residual Capacity (FRC) of the patient’s lungs, is given by:

| $v(t)=V_{iss}-\left( V_{iss}-V_{min} \right)e^{-t/{\tau_{i}}} for 0 \leq t<T_{s}$ | (6) |
| --- | --- |

where *V_iss_* is the steady state asymptote of the inspiration phase, *V_min_* is the minimum volume, which occurs at the start of inspiration, and *T_s_* is the time at which the ventilator switches from high to low pressure (Figure A1). During expiration the volume is given by:

| $v(t)=V_{ess}+\left( V_{max}-V_{ess} \right)e^{-({t-T_{s})}/{\tau_{e}}}\mathrm{for}T_{s} \leq t<T$ | (7) |
| --- | --- |

where *V_ess_* is the steady state asymptote of the expiration phase, *V_max_* is the maximum volume, which occurs at the start of expiration, and *T* is the period for the full breathing cycle. These functions are illustrated in Figure A1.

The steady state volumes are given by the product of compliance and pressure:

| $V_{iss}=C_{i}P_{i} \mathrm{and} V_{ess}=C_{e}P_{e}$ | (8) |
| --- | --- |

where *P_e_* is the low pressure setting of the ventilator (PEEP), and *P_i_*

is the high pressure used for inspiration.

Maximum volume is reach at the end of inspiration, i.e. at *t = T_s_*, and minimum volume is reached at the end of expiration, i.e. at *t = T*, so (6) and (7) can be written at these particular times as:

| $V_{max}=V_{iss}-\left( V_{iss}-V_{min} \right)e^{-{T_{s}}/{\tau_{i}}}$ | (9) |
| --- | --- |
| $V_{min}=V_{ess}+\left( V_{max}-V_{ess} \right)e^{-({T-T_{s})}/{\tau_{e}}}$ | (10) |

Define the following coefficients:

| $a=C_{i}(1-b)$ | (11) |
| --- | --- |
| $b=e^{-{T_{s}}/{\tau_{i}}}$ | (12) |
| $c=C_{e}(1-d)$ | (13) |
| $d=e^{-({T-T_{s})}/{\tau_{e}}}$ | (14) |


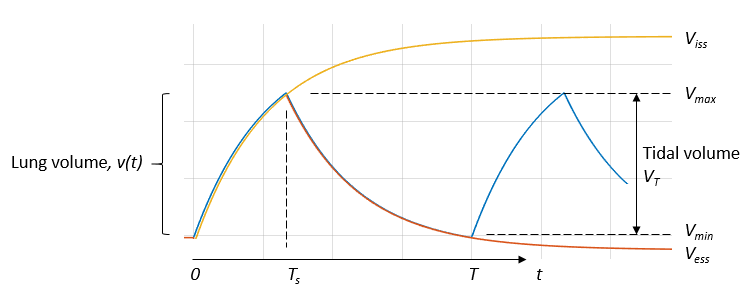


Figure A1. Lung volume waveform variable definitions

Thus the minimum and maximum volumes are related to the ventilator pressures by:

| $\left[ \begin{matrix} P_{i} \\ P_{e} \end{matrix} \right]=\mathbf{K}\left[ \begin{matrix} V_{max} \\ V_{min} \end{matrix} \right]$ | (15) |
| --- | --- |
| $\mathbf{K}=\left[ \begin{matrix} \frac{1}{a} & \frac{-b}{a} \\ \frac{-d}{c} & \frac{1}{c} \end{matrix} \right]$ | (16) |

So the maximum and minimum volume and tidal volume (*V_T_*) can be calculated:

| ${\left[ \begin{matrix} V_{max} \\ V_{min} \end{matrix} \right]\mathbf{=K}}^{\boldsymbol{-1}}\left[ \begin{matrix} P_{i} \\ P_{e} \end{matrix} \right]$ | (17) |
| --- | --- |
| $V_{T}\boldsymbol{=}V_{max}-V_{min}$ | (18) |

Equation (17) is equivalent to:

| $V_{max}=\frac{aP_{i}+bcP_{e}}{1-bd}$  $V_{min}=\frac{a{dP}_{i}+cP_{e}}{1-bd}$ | (19) |
| --- | --- |

*Calculating inspiration restrictor resistance*

The resistance required to achieve a specified tidal volume $\tilde{V}_{T}$ can be found. From (10), (14), and (18):

| $\tilde{V}_{max}=\frac{\tilde{V}_{T}+(1-d)V_{ess}}{1-d}$ | (20) |
| --- | --- |

Hence $\tilde{V}_{min}$ can be found from (18), and the new inspiration time constant can be found from (9):

| $\tilde{\tau}_{i}=-T_{s}/ln\left( \frac{V_{iss}-\tilde{V}_{max}}{V_{iss}-\tilde{V}_{min}} \right)$ | (21) |
| --- | --- |

From (5), the additional resistance, $R_{r}$, in the inspiration line can be found:

| $R_{r}=\frac{\tilde{\tau}_{i}-R_{i}C_{i}}{C_{vi}+C_{i}}-R_{vi}$ | (22) |
| --- | --- |

Alternatively, if the original tidal volume is required to be maintained but with an increased ventilator pressure *P_i_*, equation (21) can be used with a new value for the end-inspiratory steady state volume *V_iss_* given by (8), and (22) can be again used to find the restrictor resistance for the new inspiratory time constant.

As this model does not need a time-marching numerical solution, results can be determined by calculations in, for example, a spreadsheet. A view of such an implementation is given in Appendix B.
