# Supplementary figures and images for: A simple method to estimate flow restriction for dual ventilation of dissimilar patients: The BathRC model
Source: PLoS One. 2020 Nov 16;15(11):e0242123. doi: 10.1371/journal.pone.0242123 (PMC7668571; doi:10.1371/journal.pone.0242123)

**Appendix B**

A snapshot of a flow restrictor sizing spreadsheet


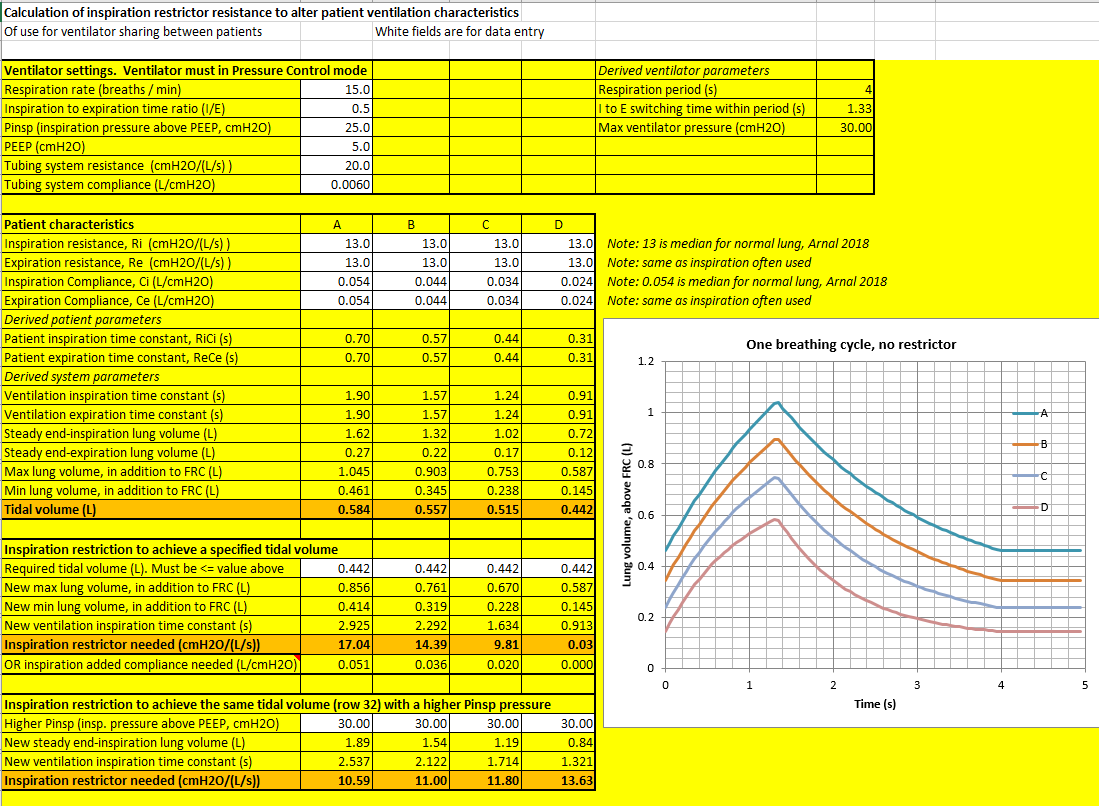

Supplement: S2 File — Appendix B. (DOCX) [file pone.0242123.s002.docx]
