# Supplementary material for: A simple method to estimate flow restriction for dual ventilation of dissimilar patients: The BathRC model
Source: PLoS One. 2020 Nov 16;15(11):e0242123. doi: 10.1371/journal.pone.0242123 (PMC7668571; doi:10.1371/journal.pone.0242123)
Supplement: Attachment — Submitted filename: ResponseToReviewersJB.docx [file pone.0242123.s003.docx]

PONE-D-20-16429
A Simple Method to Estimate Flow Restriction for Dual Ventilation of Dissimilar Patients: The BathRC Model
PLOS ONE

We would like to thank the reviewers and editor for their careful review and comments. Please find responses to each comment.

**Editorial Comments**

1. Please ensure that your manuscript meets PLOS ONE's style requirements, including those for file naming. The PLOS ONE style templates can be found at

[https://journals.plos.org/plosone/s/file?id=wjVg/PLOSOne_formatting_sample_main_body.pdf](https://eur01.safelinks.protection.outlook.com/?url=https%3A%2F%2Fjournals.plos.org%2Fplosone%2Fs%2Ffile%3Fid%3DwjVg%2FPLOSOne_formatting_sample_main_body.pdf&data=02%7C01%7Crg433%40bath.ac.uk%7Cd203bcf25bc246f00fa108d866d82476%7C377e3d224ea1422db0ad8fcc89406b9e%7C0%7C0%7C637372425790021243&sdata=dH2stdjCyEM1YR9UHA4AKVJhE1x3Kg9YJjGj6hNVXGE%3D&reserved=0) and

[https://journals.plos.org/plosone/s/file?id=ba62/PLOSOne_formatting_sample_title_authors_affiliations.pdf](https://eur01.safelinks.protection.outlook.com/?url=https%3A%2F%2Fjournals.plos.org%2Fplosone%2Fs%2Ffile%3Fid%3Dba62%2FPLOSOne_formatting_sample_title_authors_affiliations.pdf&data=02%7C01%7Crg433%40bath.ac.uk%7Cd203bcf25bc246f00fa108d866d82476%7C377e3d224ea1422db0ad8fcc89406b9e%7C0%7C0%7C637372425790021243&sdata=zYtJqot5iK%2FIdW9cKykHyKLCLsWeJsINm4SXdqTz9eQ%3D&reserved=0)

**RESPONSE:** changes made as requested

2. Thank you for stating the following in the Competing Interests section:

"The authors have declared that no competing interests exist."

We note that one or more of the authors are employed by a commercial company: BMI Bath Clinic,.

2.1. Please provide an amended Funding Statement declaring this commercial affiliation, as well as a statement regarding the Role of Funders in your study. If the funding organization did not play a role in the study design, data collection and analysis, decision to publish, or preparation of the manuscript and only provided financial support in the form of authors' salaries and/or research materials, please review your statements relating to the author contributions, and ensure you have specifically and accurately indicated the role(s) that these authors had in your study. You can update author roles in the Author Contributions section of the online submission form.

Please also include the following statement within your amended Funding Statement.

“The funder provided support in the form of salaries for authors [insert relevant initials], but did not have any additional role in the study design, data collection and analysis, decision to publish, or preparation of the manuscript. The specific roles of these authors are articulated in the ‘author contributions’ section.”

If your commercial affiliation did play a role in your study, please state and explain this role within your updated Funding Statement.

2.2. Please also provide an updated Competing Interests Statement declaring this commercial affiliation along with any other relevant declarations relating to employment, consultancy, patents, products in development, or marketed products, etc.

Within your Competing Interests Statement, please confirm that this commercial affiliation does not alter your adherence to all PLOS ONE policies on sharing data and materials by including the following statement: "This does not alter our adherence to  PLOS ONE policies on sharing data and materials.” (as detailed online in our guide for authors [http://journals.plos.org/plosone/s/competing-interests](about:blank)) . If this adherence statement is not accurate and  there are restrictions on sharing of data and/or materials, please state these. Please note that we cannot proceed with consideration of your article until this information has been declared.

Please include both an updated Funding Statement and Competing Interests Statement in your cover letter. We will change the online submission form on your behalf.

Please know it is PLOS ONE policy for corresponding authors to declare, on behalf of all authors, all potential competing interests for the purposes of transparency. PLOS defines a competing interest as anything that interferes with, or could reasonably be perceived as interfering with, the full and objective presentation, peer review, editorial decision-making, or publication of research or non-research articles submitted to one of the journals. Competing interests can be financial or non-financial, professional, or personal. Competing interests can arise in relationship to an organization or another person. Please follow this link to our website for more details on competing interests: [http://journals.plos.org/plosone/s/competing-interests](about:blank)

**RESPONSE:** The requested changes have been made.

3. We note that you have stated that you will provide repository information for your data at acceptance. Should your manuscript be accepted for publication, we will hold it until you provide the relevant accession numbers or DOIs necessary to access your data. If you wish to make changes to your Data Availability statement, please describe these changes in your cover letter and we will update your Data Availability statement to reflect the information you provide.

**RESPONSE:** The DOI has now been assigned, it is 10.15125/BATH-00816, this has been added at the appropriate point.

**Reviewers' comments:**

Reviewer #1: The COVID-19 pandemic is a global crisis, and it has renewed the interest of Dual Patient Ventilation. The authors constructed a simple model namely BathRC model. They validated the model by using a clinical ventilator connected to two test lungs, and proved that the model can be used to estimate required flow restriction for DPV. The manuscript presents an interesting work in estimation of flow restriction for DPV. The present study must have wide interests of readers, and the paper is well prepared. Its topic is prettily fitting the aims of PLOS ONE. I recommend it to be accepted for publication after the following minor-issues to be addressed.

1. Page 7 lines 147-149, the authors stated that “For each flow restrictor, all pressure drop measurements were plotted against the mean flowrate (Q m ),…A bi-square weighted robust least squares fitting method”. Why did the authors selected such the function to fit the data? Would the authors give more details about the reason of choosing the fitting function?

**RESPONSE:** The bi-square weighting fitting method was used for outlier rejection. The quadratic function represents what is seen with a combination of turbulent and laminar losses in a flow. The constant term in the quadratic is manifest when there is a spring-operated valve in the flow path.

We have now clarified the reasons for using the bi-square fitting method and for the choice of the quadratic function in the manuscript.

2. The authors validated the new model by using two test lungs. Is there any different between test lungs and human lungs? Could the model exactly be used for the real clinical trial? Please explain it, which does not weaken merits of the new model.

**RESPONSE:** The test lungs provide a safe and repeatable way to test the ventilation system and have compliances and volumes with the typical adult range. There will be variability between human subjects, however we believe the model should be able to predict the behaviour of human lungs.

Reviewer #2: The authors have done a much more in-depth experimental study than previous papers on this topic. They have also derived a very simple model and non-iterative equation that helps with coming up with a very good estimate of the amount of flow resistance that should be added to ensure that each patient receives the appropriate flow.

I haven't done much experimental work on this topic, so don't really feel I can comment on that part in detail, but they seem to me to have done a sufficiently rigorous piece of work. The experimental results are in excellent agreement with the model.

I can, however, comment on the derivation in supplementary material S1.doc, since this uses familiar circuit theory that most engineers should be able to understand. The qualitative structure of the final equation (22) is correct. However, I am a little bit uneasy with the way the derivation ignored the 2nd order term going from (3) to (4) in an arguably hand-wavy manner- there is no accompanying quantitative analysis done to justify this or citation to a paper where this was done. I would have preferred a derivation which would just state upfront that a 1st order model will be assumed, because of experimental observations, and then validate the 1st order model, which is in pretty good agreement anyway. I suspect one would get a very similar-looking equation as in (22), with slight differences in notation. However, all quantitative results and conclusions will still be the same. I therefore strongly encourage the authors to consider this in their revision.

Related questions that could have been explored, but weren't, are
(i) whether the second order model is always over-damped and
(ii) what the possible range of errors could be if one were to approximate the 2nd order dynamics with a 1st order model.
However, this is probably the topic for another paper - I wouldn't be surprised if this has already been done elsewhere in the circuit theory or lung modelling literature? Perhaps they would like to comment on this in their revision?

**RESPONSE:**

Reviewer 2 asks that a justification be given in the assumption made to go from Eqn. (3) to Eqn. (4). This has been provided, using nominal parameter values from the experimental cases to give an indication of the order of magnitude of the errors introduced by this assumption. Further suggestions in (i) and (ii) are, as the reviewer says, too large a topic for inclusion in full here, but these have been addressed to some extent: for (i) we have provided the answer but not the proof; and for (ii) we have provided a quantification of nominal values but not a full study of the range of these values. This is all included in the short paragraph newly introduced after Eqn. (4). For the reviewer’s scrutiny, the full derivations are provided below.

**Paragraph included after Eqn. (4)**

It can be shown that the system in Eqn. 3 is always overdamped for physically possible values of $R$, $C$, $R_{v}$ and $C_{v}$ (i.e. positive values). The second order system is thus comprised of two first-order lags. Using the nominal parameter values from the experiments below, these lags have time constants of 1.25 s and 0.0296 s. For comparison, the first order system in Eqn. 4 has a single time constant of 1.28 s. This value is very close to one of the second order time constants, while the remaining time constant is too small to have a significant contribution at the timescales considered. Transfer functions for the first-order system and the second-order system differ by less than 2% in amplitude at the nominal respiration rate of 0.25 Hz.

**Damping in second order system**

The second order system in eqn. 3 is always over-damped, and this is proven as follows. From eqn. 3, the damping ratio for the system is

$$\zeta=\frac{RC+R_{v}C_{v}+R_{v}C}{2\sqrt{RCR_{v}C_{v}}}$$

Substituting $\phi_{R}=R_{v}/R$ and $\phi_{C}=C_{v}/C$ gives

$$\zeta=\frac{1+\phi_{R}\phi_{C}+\phi_{R}}{2\sqrt{\phi_{R}\phi_{C}}}.$$

For an over-damped response, $\zeta-1>0$, or

$$\frac{1+\phi_{R}\phi_{C}+\phi_{R}-2\sqrt{\phi_{R}\phi_{C}}}{2\sqrt{\phi_{R}\phi_{C}}}>0.$$

For positive, real values of $\phi_{R}$ and $\phi_{C}$ (i.e. physically possible values) the denominator can be eliminated to give the criterion for an over-damped response as

$$1+\phi_{R}\phi_{C}+\phi_{R}-2\sqrt{\phi_{R}\phi_{C}}>0$$

or equivalently

$$\left( 1+\phi_{R}\phi_{C}+\phi_{R} \right)^{2}-4\phi_{R}\phi_{C}>0.$$

Defining

$$f\left( \phi_{R},\phi_{C} \right)=\left( 1+\phi_{R}\phi_{C}+\phi_{R} \right)^{2}-4\phi_{R}\phi_{C}$$

then setting $\phi_{R}$ and $\phi_{C}$ to zero gives $f\left( \phi_{R},\phi_{C} \right)=1$, a positive result. So if an under-damped configuration exists for positive $\phi_{R}$ and $\phi_{C}$ then the function $f\left( \phi_{R},\phi_{C} \right)$ would need to transition from positive to negative and there would need to be a solution that satisfies $f\left( \phi_{R},\phi_{C} \right)=0$, or

$$1+2\phi_{R}+{\phi_{R}}^{2}{\phi_{C}}^{2}+2{\phi_{R}}^{2}\phi_{c}+{\phi_{R}}^{2}-2\phi_{R}\phi_{C}=0.$$

Rearranging gives

$${\phi_{R}}^{2}\left( {\phi_{C}}^{2}+2\phi_{c}+1 \right)+\phi_{R}\left( 2-2\phi_{C} \right)+1=0$$

and solving for $\phi_{R}$ gives

$$\phi_{R}=\frac{\phi_{C}-1\pm\sqrt{-4\phi_{c}}}{{\phi_{C}}^{2}+2\phi_{c}+1}.$$

A real valued solution for $\phi_{R}$ only exists for non-positive values of $\phi_{C}$, which are physically impossible, so it is concluded that $\zeta-1>0$ for all physically possible values of $R$, $C$, $R_{v}$ and $C_{v}$. The second order system in eqn. 3 will therefore always be overdamped.

**Approximation to first order system**

The second order system in eqn. 3 has the characteristic equation

$${RCR_{v}C_{v}s}^{2}+\left( RC+R_{v}C_{v}+R_{v}C \right)s+1=0$$

which yields roots at

$$s=-\frac{RC+R_{v}C_{v}+R_{v}C}{2RCR_{v}C_{v}}\pm\sqrt{\left( \frac{RC+R_{v}C_{v}+R_{v}C}{2RCR_{v}C_{v}} \right)^{2}-\frac{1}{RCR_{v}C_{v}}}.$$

The system is over-damped so will have two real roots corresponding to the time constants of two first-order lags. Neglecting the second order term, the root of the resulting first order system is

$$s=\frac{-1}{RC+R_{v}C_{v}+R_{v}C}.$$

The first order system is a good approximation to the second order system if: (a) one of the two second order poles is close to the first order pole, (b) there is good separation between the two second order poles, and (c) the operating frequency is well below that of the highest second-order pole.

Using the nominal values from the experiment in the first row of Table 2, the time constants for the first order system are 0.0296 s and 1.25 s, and the time constant for the second order system is 1.28 s. The first order system matches the slowest mode of the second order system to within 2.4%, satisfying criterion (a). The two modes of the second order system are separated by a factor of 42, satisfying criterion (b). The operating frequency is 15 breaths/min or 0.25 Hz, which is well below the 5.4 Hz of the fastest second order mode, satisfying criterion (c).

At the operating frequency of 15 breaths/min the amplitude and phase response of the second order system is -1.62$\times$10^-7^ m^3^/Pa and -65.6^o^ while that of the first order system is -1.59$\times$10^-7^ m^3^/Pa and -63.5^o^, giving an amplitude difference of less than 2% and a phase difference of only 2.1^o^. The first order system thus provides a good approximation to the second order system in the context of this study.
